# Meta-analysis on the Therapeutic State of Hypertensive Population in Japan: Focusing on the Impact of New Diagnostic Criteria of Japanese Guideline for the Management of Hypertension 2000

**DOI:** 10.2188/jea.12.112

**Published:** 2007-11-30

**Authors:** Toshihiko Hasegawa, Yoko Hori, Hiroyuki Sakamaki, Kazuo Suzuki

**Affiliations:** 1 Department of Health Care Policy, National Institute of Health Services Management.; 2 Research Department, Institute for Health Economics and Policy.; 3 Department of Epidemiology, Research Institute for Brain and Blood Vessels Akita.

**Keywords:** hypertension, meta-analysis, national nutrition survey, national survey of circulatory disorders, guidelines for the management of hypertension in Japan (JSH2000)

## Abstract

A Meta-analysis on the therapeutic state of hypertensive population in Japan is performed by the three nation-wide governmental surveys focusing on the impact of new diagnostic criteria described in the Guidelines for the Management of Hypertension in Japan 2000. These surveys are the National Survey of Circulatory Disorders, National Nutrition Survey and Patient Survey in 1990. The meta-analysis approach is used to evaluate the validity and reliability of these three national data sets, particularly the National Nutrition Survey. The population with history of hypertensive treatment and without previous diagnosis was calculated using the old and new diagnostic criteria. The results of three national surveys are fairly consistent. National Nutrition survey can be used to monitor the overall therapeutic status of Japanese population if the definition is considered judiciously. The impact of new diagnostic criteria is extensive as demonstrated by the results of the analysis on the National Nutrition Survey of 1999. The hypertensive population doubled and one half of the Japanese population over the age of 30 is now defined as hypertensive. A policy to manage this newly diagnosed hypertensive population is urgently needed to lessen the burden on Japanese health care system.

## INTRODUCTION

Hypertension is an important disease since it is a major contributing risk factor to other circulatory disease and health care cost^1^^,^^2^^)^. In Japan, in particular stroke was the No.1 killer in the past and now has become the largest cause of elderly disability. Health care costs of hypertension treatment can be considered as a good investment if hypertension is controlled well. It is important to evaluate the therapeutic status of the hypertensive population. But the estimation of therapeutics status by different nation-wide government surveys is conflicting^3^^-^^5^^)^. The objective of this paper is to estimate the therapeutic status of hypertensive population in Japan by Meta-analysis comparing two different kind of survey, a population based survey such as National Nutrition Survey (Note 1), National Survey of Circulatory Disorder (Note 2) and medical facility based surveys, like Patient Survey (Note 3). Application of the results of this study is to authenticate the validity and reliability of National Nutrition Survey and Patient Survey, since National Survey of Circulatory Disorders is done only once every ten years. National Nutrition Survey and Patients Survey on the other hand, is available for continuous monitoring because they are done every year and every three years. Then the impact of new diagnostic criteria of Guidelines for the Management of Hypertension in Japan 2000 (Note 4, Table 2) is evaluated using National Nutrition Survey of 1999^6^^-^^8^^)^.

## MATERIALS AND METHODS

### 1. Meta-analysis between National Survey of Circulatory Disorders and National Nutrition Survey

The therapeutic status measured by National Survey of Circulatory Disorders and National Nutrition Survey is organized around two different principles. The National Survey of Circulatory Disorders is based upon a visit to health care facility, and the National Nutrition Survey is based on drug treatment. The estimation of hypertensive population in both surveys is calculated through the prevalence rate in 5 year-age and sex groups and multiplied by each group population.

From the National Survey of Circulatory Disorders, data were divided by the presence of past history of hypertension. For the no past history group, hypertensive population was identified by using the old criteria i.e. the systolic pressure of more than 160 mm Hg or the diastolic pressure more than 95 mm Hg (Note 4, Table 1). For the population with a previous history, three groups, namely, no visit, one visit over more than a one month period, and one visit in less than one month period, and others probably indicating irregular visit were identified.

For National Nutrition Survey, data is classified according to drug treatment status. No drug treatment groups were also classified by blood pressure, systolic pressure of more than one 160 mm Hg, the diastolic pressure more than 95 (Note 4, Table 1). For the population with history of medication, three groups, namely those that stopped taking medicine, those taking it occasionally, those taking it daily, were identified. Nation-wide estimate is calculated by similar method as the case of National Survey of Circulatory Disorders.

### 2. Meta-analysis between National Survey of Circulatory Disorders and Patient Survey

For National Survey of Circulatory Disorders 1990, patient with previous treatment is selected and classified into three groups, namely no current visit to medical facility, visit once more than one month, and visit once less more than one month. The data were classified into five-year age group and sex. The ratio was calculated for each group and multiplied by the general population for each segment to estimate the total number of patients.

For Patient Survey, the 1990 database was used. For each five-year age group and sex group, total patient were calculated using the following formula used by Japanese Government Statistics^9^^)^.

$$\begin{array}{rl} \text{Total patient} & =\text{In-patient number}+\text{Out-patient number of}\\ & \;\text{one day visit}\times \frac{6}{7}\\ & \;\text{Visit interval (day)} \end{array}$$

Main and sub diagnosis of hypertension, visit interval more than one month are included for patients. 95% confidence interval (CI) was calculated according to sample size. Estimates of those two databases are compared by five-year age and sex groups.

### 3. The Estimate of the Impact by New Diagnostic Criteria

By using the National Nutrition Survey 1999 the same calculation was done to estimate the total number of patients by age and sex group. For population without any previous medication, the three categories of population are estimated by age and sex group using new diagnostic criteria. One group composed of those with blood pressure values of more than systolic 160 or diastolic 95 (Note 4, Table 1). Next was the group of the newly diagnositic hypertensives. Lastly there was a group for the “high normal”. Nation wide population was calculated for high normal, new mild hypertensive and traditional undiagnosed hypertensive population. Among treated groups there were those that were taking medication occasionally or daily. Control fraction of treated population were calculated by using 5 year age and sex group fraction multiplied by each segment population.

## RESULTS

### 1) The Estimate of Hypertensive Population by National Survey of Circulatory Disorders 1990 and National Nutrition Survey 1990

**Figure 1.**
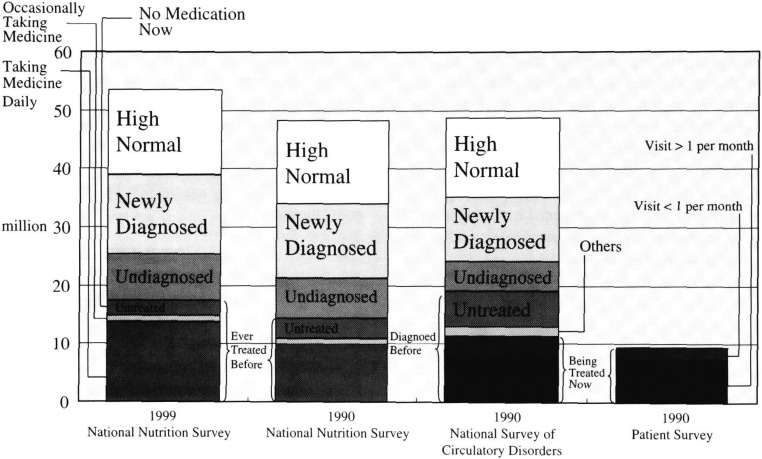
Hypertensive population estimate by different classifications and surveys.

According to the 1990National Survey of Circulatory Disorders, population ever treated for hypertension is 18,792,000 including those under admission numbering 16,000 (Figure 1). Outpatient visiting population is 11,151,000. Population with no visit is 6,000,000. Others is 1,627,000. Among the population without previous visit to a medical facility 5,218,000 are hypertensive when using the old diagnostic criteria. Another 11,029,000 are classified as hypertensive when using the new diagnostic criteria. 13,702,000 are normal high when the new diagnostic criteria are applied. Adding these numbers up, there are 22,953,000 hypertensives in the population using the old criteria and 34,982,000 according to the new criteria. According to the National Nutrition Survey 1990, population ever treated by medication is 14,187,000, including those taking daily medication at 9,843,000 and those taking medication occasionally at 932,000 and those that have stopped medication at 3,413,000. Among the population with no previous history of medication 7,149,000 are hypertensive based on old diagnostic criteria, and 12,631,000 are hypertensive based to the new diagnostic criteria with another 14,412,000 for normal high.

### 2) Comparative Estimate of Hypertensive Population under Medical Care by National Survey of Circulatory Disorders 1990 and the Patients Survey 1990

**Figure 2.**
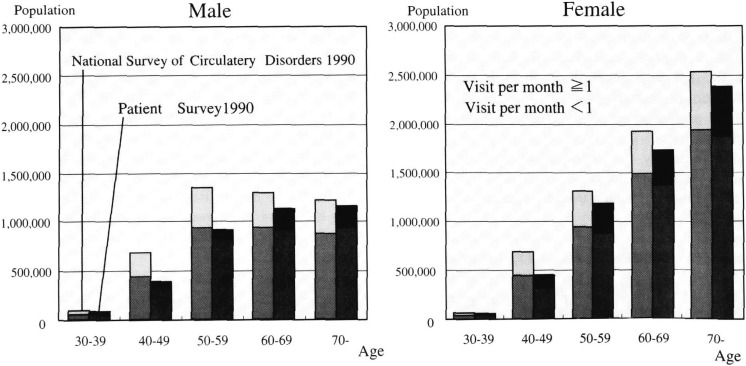
Comparative estimate of hypertensive patients by 10 year age, sex group and visit per month.

Estimate using 1990 Patients Survey is 9,422,000 (95%C.I. 9,177,000 - 9,667,000) which is 1,729,000 (15.5%) smaller than the estimate by the 1990 National Survey of Circulatory Disorders. But most of the difference comes from the patients visiting less than once a month, that is, 1,115,000 (95%C.I. 7,853,448 - 13,240,288) by age and sex group (Figure 2). The largest discrepancy is among males aged between 42-60 years visiting medical care facility less than once a month. This discrepancy is generally smaller for females.

### 3) Estimation of the Impact of New Diagnostic Criteria Using during the National Nutrition Survey of 1999

**Figure 3.**
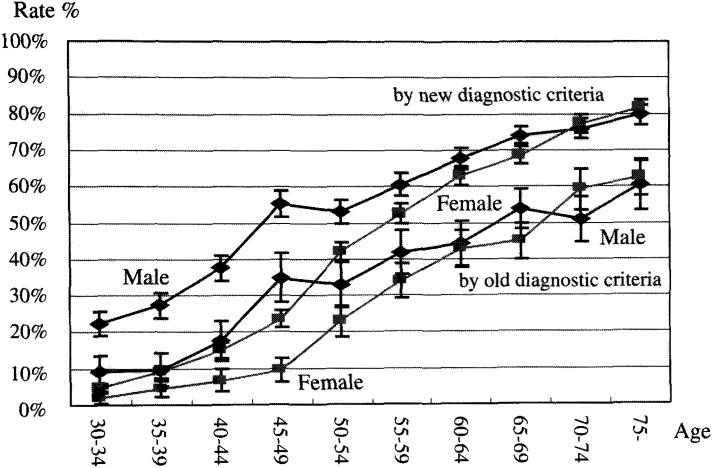
Hypertensive population rate by 5 years and sex group, by old and new diagnostic criteria with 95% confidence interval.

**Figure 4.**
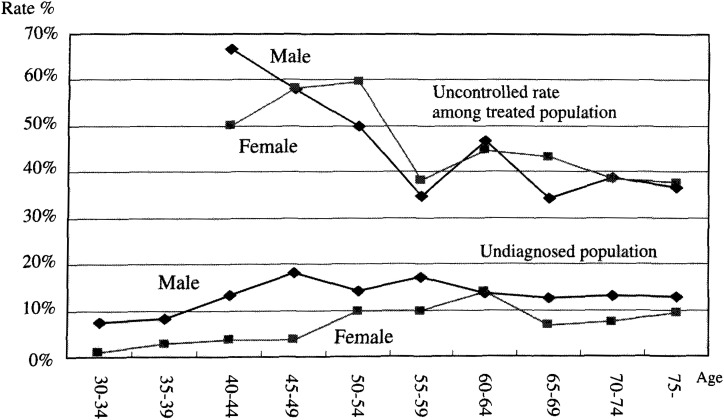
Undiagnosed and uncontrolled rate by 5 years and sex group, by old diagnostic criteria.

Same methods as described above were used to estimate the patients ever treated previously with medication in the hypertensive population without previous drug treatment, using the old and new criteria. Sex and five year age groups were used to calculate. Population ever treated was 17,195,000 including those already stopped medication at 2,498,000 (Figure 1). Among the population never treated with drugs, 8,111,000 were hypertensive based on the old diagnostic criteria. Another 21,724,000 were hypertensive based on the new criteria with an additional 14,675,000 as “high normal”. When all of these numbers are added together, the hypertensive population totals 25,306,000 applying the old criteria (31.0% of total population age over 30) and 47,030,000 (47.7% of total population age over 30) applying the new criteria. If the “high normal” are also added, hypertensive and high normals amount to 53,593,577 (65.7% of total population age over 30). The population fraction of hypertensive population by sex and age group is shown in the Figure 3. The uncontrolled blood pressure fraction among treated hypertensive population is calculated and is shown (Figure 4).

## DISCUSSION

### 1) Meta-analysis for Validity and Reliability of Different Data Sets

Discrepancy of estimated number of patients under current treatment between national cardiovascular diseases survey and National Nutrition Survey is very small 0.38 million in 1990 (3.4% of treated population) (Figure 1). This can be due to a random error or a non-pharmacologically treated population. But discrepancy between population never treated and the population ever treated is 46 million that is significant. It is possible that National Survey of Circulatory Disorders included currently no visiting population. 30% of population with previous diagnostic history of hypertension but currently not visiting a medical facility stopped medication or at least has never taken medication. So about 4 million patients could have been white-coat hypertension or had been controlled by no-pharmacological treatment since about half of this population is normotensive. On the other hand, there is the real hypertensive population because hypertension is a life long disease. Undiagnosed population according to the National Survey of Circulatory Disorders is 5.2 million. This is 7.1 million according to the National Nutrition Survey. The discrepancy is 1.7million but it is probably due to hypertensive fraction of the population who is not visiting to medical care facility. Total was 24.0 million vs. 22.5 million hypertensive population when using the old criteria. This discrepancy is only 1.5 million (6%). However, when the new criteria is used, the differences is small 0.6 million. The undiagnosed fraction of hypertensive population is 21.8% by National Survey of Circulatory Disorders and 31.8% by National Nutrition Survey. This is a 10% difference. Therefore, the total number of the hypertensive population is not different according to the 2 surveys, but the undiagnosed population fraction is different due to the application of different definitions. Caution is therefore required to interpret the undiagnosed fraction when National Nutrition Survey is used. About one third of such a population was once diagnosed as hypertensive but has not been followed up.

For the patients who have been under treatment, the discrepancy between National Survey of Circulatory Disorders and Patient Survey is 1.6million (16.4%). Most of this discrepancy can be explained by the discrepancy in middle age group of the male hypertensive population (Figure 2). The discrepancy could be due to a random error but also the bias due to the sampling methods of survey. Those middle age males are usually the working population. If the clinic at working place is excluded for Patient Survey, the number could be underestimated. But on the other hand, the Patients Survey is supposed to be randomized at the clinic level^10^^)^. The other possibility is the recall-reporting bias of National Survey of Circulatory Disorders because it is a self-reported survey done by the medical professions as patients usually responds favorably. Nevertheless, the most likely scenario is the problem of compliance, as middle aged workers tend to attend medical care facility very infrequently^5^^)^. The patients treated with medication have an overall uncontrolled rate of 46.5%, but can be as high as 68% in males aged 55 to 49 years old (Figure 4). In the 1999 survey, the controlled rate was brought down in numbers, particularly in the old age group. However when the new diagnostic criteria were applied, the uncontrolled rate became 79.4%. Since the new guidelines had not been developed until year 2000, target for treatment then was not the new criteria.

### 2) Impact of New Diagnostic Criteria

Hypertensive population using the old criteria in the 1999 survey was estimated at 25.3 million cases. This is an increment of 2.8 million since 1990 (Figure 1). Undiagnosed population increased to 8.1 million with a 1 million increment since the 1990 survey. The fraction of undiagnosed patients is about 30% using the old criteria and this was not different from the 1990 survey. But caution has to be exercised because this fraction could be smaller depending on the definition. Applying the new criteria, another 21.7 million were included in the hypertensive population. This totals to 47.0 million, which is 47.7% of population over 30 years of age. The application of the new criteria almost doubles the hypertensive population and now one out of every two Japanese is considered to be hypertensive. If the “high normal” population is also included, then 65% of population is at some risk according to the new diagnostic criteria. The fraction of hypertensive population increases as population ages. The males are always higher than females and at the age over 75, the hypertensive population is about 80% when using the new criteria and about 60 % when using even the old criteria. For estimating the therapeutic status using the old criteria, 8.1 million people are undiagnosed and another 6.3 million people diagnosed previously as hypertensive are not under control (Figure 3). The therapeutic strategy of expanding diagnostics criteria by JSH 2000 leads to the inclusion of 21 million new patients. This may not be practical because the large hypertensive population by old criteria still has not been controlled well. Lastly, white-coat hypertension i.e. transient hypertension upon meeting the medial profession is well known^11^^-^^15^^)^. 10 to 40% of hypertension could be in this category. At least 10% of the 8 million undiagnosed hypertensive population using old criteria and another 21 million using new criteria could be actually an overestimation, because the measurement of blood pressure for National nutrition survey is only at one occasion not particularly time. At least 10% of the 15 million people who are treated with medication by National nutrition survey 1990 population might have been treated unnecessarily. On other hand, false negative blood pressure values have been reported as well^16^^)^. To examine those false (positive and negative) in value requires continuous blood pressure monitoring or home based blood pressure assessments using better measurement methods. It may be difficult to use those methods for nation-wide study but the result of a pilot study in certain areas could be useful to adjust a major survey results^17^^)^.

## CONCLUSION

By meta-analysis of three national data set for hypertension, the estimate is consistent provided definition of classification has been taken into account. Discrepancy between National Survey of Circulatory Disorders and National Nutrition Survey seems to be due to the populations who are diagnosed hypertensive but not treated currently. However, the overall hypertensive population is quite similar. National Nutrition Survey can be used for annual follow up and evaluation. Discrepancies between National Survey of Circulatory Disorders and Patients Survey are mainly due to middle-aged male hypertensive population. The reason could be random errors and recall bias. A detailed study is required to reveal the main reason. Nevertheless the overall trend is consistent and the Patient Survey can be very good basis to analyze treatment behavior of hypertensive patients. The impact of the new diagnostic criteria is tremendous. The number of patients becomes double and more than half of the population of aged over 30 in Japan is labeled as hypertensive. The undiagnosed population increases from 22.3% up to 50%. Blood pressure controlled fraction of treated patients decreased from about 60% to 20%. This change will add a large new treatment burden to medical facility and financial burden to social insurance.

## ACKNOWLEDGMENT

The Authors would like to thank the following persons for kind discussion and suggestion.

Syunroku Baba, National Cardiovascular Center. Hirotsugu Ueshima, Department of Health Science, Shiga University of Medical Science. Ikao Saito, Health Center, Keio University. Yutaka Imai, Department of Clinical Pharmacology and Therapeutics, Tohoku University Graduate School of Medicine and Pharmaceutical Science. And the technical support Kunichka Matsumoto and Yoshihiro Kitamura, National Institute of Health Services Management.

This paper is funded by Grants-in-Aid for Scientific Research from the Ministry of Health, Labor and Welfare, Research on the technology assessment of hypertension arrangement (No. H12-Iryo-002) and Research on the promotion and evaluation of Healthy Japan 21 (No. H12-Kenko-003).

## Notes

### 1. National Nutrition Survey

300 living area was randomly selected based upon area of the Comprehensive Survey of Living Condition of the People on Health and Welfare. Interview, blood pressure anthropometrical measurement and bloody chemistry test were performed on each person. Blood pressure was measured by auscultation using a mercury sphygmomanometer. As a rule, measurements were taken from the right arm with the subjects. If the first measurement values were outside the normal range, the measurements were repeated. Sample number is 17,986 in 1990 and 12,763 in 1999. Survey stated in 1951 and to include hypertension treatment status after 1986.

### 2. The National Survey of Circulatory Disorders

The National Survey of Circulatory Disorders was done about every ten years 1971, 1980,1990, 2000; the most recent available data was 1990. A Nation wide random sample aged over 30 was taken from the chosen 300 living areas based upon the areas defined by the Comprehensive Survey of Living Condition of the People on Health and Welfare. New interview questions was added to ask the past history and treatment of circulatory disease in addition to the nutrition survey. Sample size was 10,956 in 1990.

### 3. National Patient Survey

Patient Survey has been done since 1953. After 1984, survey was done every 3 years with larger sample size. Particular, after 1993, sample was increased up to 70%. Survey consists of two parts. First part is to measure the prevalence of inpatient and outpatient by one-day survey. Second part is as the discharge survey during the month of September. 20% of hospitals and 5% of clinics were selected using a random sample of prefecture in 1990. The survey consisted of age and sex, visit interval, age and sex, main diagnosis and sub diagnosis.

### 4. Diagnostic Criteria

The old diagnostic criteria of WHO (Table 1) has been used for a long time until recently^16^^,^^17^^,^^18^^)^. Japanese Ministry of Health & Welfare and Japanese Medical Association developed the first guideline based on the WHO criteria in 1990 under the recommendation of Ministry of Health & Welfare. The Japanese Society of Hypertension developed new guideline based on new WHO criteria in 2000^19^^,^^20^^)^ and the sixth report of the joint National committee on Prevention, Deletion, Evaluation, and Treatment of high blood pressure (JNC-IV) (Table 2)^22^^)^.

**Table 1.** Old criteria by old WHO guideline.

	Systolic Pressure (mmHg)		Diastolic Pressure (mmHg)
Borderline	140-159	or	90-94

Hypertension	≧ 160	or	≧95

**Table 2.** Diagnostic criteria: JSH (2000), JNC-VI (1997), WHO/ISH (1999).

JCH Criteria (JNC VI Criteria, WHO/ISH)	JSH (2000) JNC-VI (1997)			WHO/ISH (1999)		
	
	Systolic Pressure (mmHg)		Diastolic Pressure (mmHg)	Systolic Pressure (mmHg)		Diastolic Pressure (mmHg)
High Normal (High Normal)	130 – 139	or	85 – 89	130 – 139	or	85-89
				140 – 159		90-99
Mild Hypertension (Hypertension stage1, grade1)	140 – 159	or	90 – 99	subgroup: borderline 140 – 149	or	subgroup: borderline 90 – 94

Moderate Hypertension (Hypertension stage2, grade2)	160 – 179	or	100 – 109	160 – 179	or	100 – 109
Severe Hypertension (Hypertension stage3, grade3)	≧ 180	or	≧ 110	≧ 180	or	≧ 110
